# The paradigm of IL-23-independent production of IL-17F and IL-17A and their role in chronic inflammatory diseases

**DOI:** 10.3389/fimmu.2023.1191782

**Published:** 2023-08-04

**Authors:** Victoria Navarro-Compán, Luis Puig, Silvia Vidal, Julio Ramírez, Mar Llamas-Velasco, Cristina Fernández-Carballido, Raquel Almodóvar, José Antonio Pinto, Eva Galíndez-Aguirregoikoa, Pedro Zarco, Beatriz Joven, Jordi Gratacós, Xavier Juanola, Ricardo Blanco, Salvador Arias-Santiago, Jesús Sanz Sanz, Rubén Queiro, Juan D. Cañete

**Affiliations:** ^1^ Department of Rheumatology, Hospital Universitario La Paz, IdiPaz, Madrid, Spain; ^2^ Department of Dermatology, Hospital de la Santa Creu i Sant Pau, Barcelona, Spain; ^3^ Immunology-Inflammatory Diseases, Institut de Recerca de l’Hospital de la Santa Creu i Sant Pau, Biomedical Research Institute Sant Pau (IIB Sant Pau), Barcelona, Spain; ^4^ Arthritis Unit, Department of Rheumatology, Hospital Clínic and Instituto de Investigaciones Biomédicas August Pi i Sunyer (IDIBAPS), Barcelona, Spain; ^5^ Department of Dermatology, Hospital Universitario La Princesa, Madrid, Spain; ^6^ Department of Rheumatology, Hospital Universitario San Juan de Alicante, Alicante, Spain; ^7^ Department of Rheumatology, Hospital Universitario Fundación Alcorcón, Alcorcón, Madrid, Spain; ^8^ Department of Rheumatology, Complejo Hospitalario Universitario de A Coruña, Instituto de Investigación Biomédica de A Coruña (INIBIC), A Coruña, Spain; ^9^ Department of Rheumatology, Hospital Universitario de Basurto, Bilbao, Spain; ^10^ Department of Rheumatology, Hospital Universitario 12 de Octubre, Madrid, Spain; ^11^ Department of Rheumatology, Medicine Department Autonomus University of Barcelona (UAB), I3PT, University Hospital Parc Taulí Sabadell, Barcelona, Spain; ^12^ Department of Rheumatology, University Hospital Bellvitge, Instituto de Investigación Biomédica de Bellvitge (IDIBELL), Barcelona, Spain; ^13^ Department of Rheumatology, Hospital Universitario Marqués de Valdecilla, Instituto de Investigación Marqués de Valdecilla (IDIVAL), Santander, Spain; ^14^ Department of Dermatology, Hospital Universitario Virgen de las Nieves, Granada, Spain; ^15^ Instituto de Investigación Biosanitaria ibs.GRANADA, Granada, Spain; ^16^ Department of Dermatology, Facultad de Medicina, Universidad de Granada, Granada, Spain; ^17^ Department of Rheumatology, Hospital Universitario Puerta del Hierro Majadahonda, Madrid, Spain; ^18^ Department of Rheumatology, Hospital Universitario Central de Asturias, Oviedo, Asturias, Spain

**Keywords:** IL-17A, IL-17F, IL-23, spondyloarthritis, Th17 cells, MAIT cells, γδ T cells, psoriasis

## Abstract

Interleukin-17 family (IL-17s) comprises six structurally related members (IL-17A to IL-17F); sequence homology is highest between IL-17A and IL-17F, displaying certain overlapping functions. In general, IL-17A and IL-17F play important roles in chronic inflammation and autoimmunity, controlling bacterial and fungal infections, and signaling mainly through activation of the nuclear factor-kappa B (NF-κB) pathway. The role of IL-17A and IL-17F has been established in chronic immune-mediated inflammatory diseases (IMIDs), such as psoriasis (PsO), psoriatic arthritis (PsA), axial spondylarthritis (axSpA), hidradenitis suppurativa (HS), inflammatory bowel disease (IBD), multiple sclerosis (MS), and asthma. CD4^+^ helper T cells (Th17) activated by IL-23 are well-studied sources of IL-17A and IL-17F. However, other cellular subtypes can also produce IL-17A and IL-17F, including gamma delta (γδ) T cells, alpha beta (αβ) T cells, type 3 innate lymphoid cells (ILC3), natural killer T cells (NKT), or mucosal associated invariant T cells (MAIT). Interestingly, the production of IL-17A and IL-17F by innate and innate-like lymphocytes can take place in an IL-23 independent manner in addition to IL-23 classical pathway. This would explain the limitations of the inhibition of IL-23 in the treatment of patients with certain rheumatic immune-mediated conditions such as axSpA. Despite their coincident functions, IL-17A and IL-17F contribute independently to chronic tissue inflammation having somehow non-redundant roles. Although IL-17A has been more widely studied, both IL-17A and IL-17F are overexpressed in PsO, PsA, axSpA and HS. Therefore, dual inhibition of IL-17A and IL-17F could provide better outcomes than IL-23 or IL-17A blockade.

## 1 Introduction

Interleukin (IL)-17A and IL-17F are a pair of rather newly described pro-inflammatory cytokines capable of bridging the adaptative and innate immune systems. Both cytokines have a role in maintaining epithelial barrier function (skin, intestinal epithelium, gingiva, vaginal mucosa, and conjunctiva) and providing protection against pathogens (1). However, alterations in the regulation and excess of these two cytokines have pathogenetic implications in chronic immune-mediated inflammatory diseases (IMIDs), including psoriasis (PsO), psoriatic arthritis (PsA), axial spondyloarthritis (axSpA), hidradenitis suppurativa (HS), and inflammatory bowel disease (IBD) (2).

IL-17A and IL-17F are produced by cells of the innate and adaptative immune system and activate the production of inflammatory mediators such as tumor necrosis factor α (TNFα), IL-1β, IL-6, granulocyte colony-stimulating factor (G-CSF), and granulocyte-macrophage colony-stimulating factor (GM-CSF). They also induce the production of chemokines, including CXCL1, CXCL5, CCL2, and CCL7, and the expression of antimicrobial peptides (AMPs), which mediate the activation and recruitment of inflammatory cells such as neutrophils (3).

IL-23 is a cytokine particularly important in maintaining the differentiation state of Th17 cells, the best-known producer of IL-17A and IL-17F. In this context, the discovery of the IL-23/IL-17 axis prompted the development of several therapeutic strategies for autoimmune disorders and chronic inflammation (4–6). To date, both IL-23p19 and IL-12/23 inhibitors (guselkumab, risankizumab, tildrakizumab, and ustekinumab), as well as IL-17RA and IL-17As inhibitors (brodalumab, ixekizumab, and secukinumab) have been tested in several IMIDs, including PsO, PsA, axSpA, IBD and HS (**Table 1**). However, their blockade did not yield the same clinical outcomes in all IMIDs (4, 6–10). A possible explanation is tissue specificity of these cytokines (11, 12). One example is the lack of efficacy of anti-IL-12/23 and anti-IL-23p19 monoclonal antibodies (mAb) therapy in axSpA and HS, unlike IL-17A inhibitors (13–19). Studies performed in animal models provided a plausible explanation for this differential behavior as IL-23 was found to be required for the initiation but not for the maintenance of the disease (20). Conversely, the inhibition of the IL-23 provides good results in IBD (21), bringing about the approval of biological treatments whereas neutralization of IL-17A did not (22, 23). However, IL-17F suppression was effective in a colitis mouse model, indicating that IL-17A and IL-17F could have differential roles in the gut (24). Indeed, recent studies suggest that IL-17A and IL-17F could have protective and pathogenic roles in the gut, respectively (24–27). Moreover, beyond Th17 cells, innate cells and innate-like lymphocytes also produce IL-17s in an IL-23-independent manner. Several studies have stressed that in certain contexts innate cells can be a major source of IL-17A and IL-17F and that they can produce these cytokines regardless of IL-23 stimulus, playing important roles in inflammation and autoimmunity (28, 29). Clinical and pre-clinical studies in PsO and SpA suggested that targeting IL-17F in addition to IL-17A could lead to increased suppression of proinflammatory genes (30, 31), reduced migration of both adaptive and innate immune cell types (31) and reduced periosteal stem cell bone formation (32), compared with the inhibition of IL-17A alone. Consequently, dual or bispecific inhibitors, such as bimekizumab or sonelokimab, have been developed with promising results in IMIDs (33). Altogether the evidence suggests a non-linear relationship between IL-23 and the IL-17s, as well as tissue-specific functions of IL-17A and IL-17F.

**Table 1:** Available therapies targeting IL-17 and IL-23 in IMIDs.

Mechanism of action	Therapy	Approved indications*	PsO	PsA	axSpA	HS	IBD	Uveitis
Anti-IL-17A	Ixekizumab	Plaque psoriasis, psoriatic arthritis, axial spondylarthritis						
	Secukinumab	Plaque psoriasis, psoriatic arthritis, axial spondylarthritis, juvenile idiopathic arthritis						
Anti-IL-17RA	Brodalumab	Plaque psoriasis						
Anti IL-17A and IL-17F	Bimekizumab	Plaque psoriasis, psoriatic arthritis, and axial spondyloarthritis. Phase 3 clinical development completed for hidradenitis suppurativa, EMA approval pending						
Anti-IL12/23	Ustekinumab	Plaque psoriasis, psoriatic arthritis, Crohn’s disease, ulcerative colitis						
Anti-IL23p19	Guselkumab	Plaque psoriasis, psoriatic arthritis. In phase 3 clinical development for Crohn’s disease and ulcerative colitis						
	Risankizumab	Plaque psoriasis, psoriatic arthritis, Crohn’s disease. In phase 3 clinical development for ulcerative colitis						
	Tildrakizumab	Plaque psoriasis. In phase 3 clinical development for psoriatic arthritis						

axSpA, axial spondyloarthritis; HS, hidradenitis suppurativa; IBD, inflammatory bowel disease; IL, interleukin; PsA, psoriasis arthritis; PsO, psoriasis.

*The table only includes therapies that have been approved by the European Medicines Agency (EMA) or that have published phase 3 clinical development.

Green: approved or shown efficacy in phase 3 clinical trials. Red: Lack of efficacy. Yellow: Insufficient or unclear evidence.

Thus, the role of IL-17A and IL-17F in IMIDs is complex, involving the adaptative and innate immune system, and cytokine signaling beyond the IL-23/IL-17 axis. The present narrative review aims to examine the evidence about the involvement of IL-17A and IL-17F in inflammatory diseases exploring alternative innate sources and IL-23-independent signaling, which represent alternative escape routes that perpetuate the inflammatory loop and can influence treatment choice.

## 2 Biology of IL-17s and their receptors

IL-17A is considered the founding member of the IL-17 family and thus is the most studied. However, the family includes five additional members, as revealed by sequence homology studies: IL-17B, IL-17C, IL-17D, IL-17E (also named IL-25), and IL-17F (34, 35). Among the IL-17 family members, the roles and functions of IL-17A and IL-17F are intertwined partly due to their high amino acid sequence homology (around 50%) and their shared common evolution, since their corresponding genes can be found in close proximity on chromosome 6 (36). The rest of the members have much lower homology (16-30%) and are located on different chromosomes (**Supplementary Table 1**) (37, 38). IL-17s function as dimeric cytokines, as revealed by structural analysis (39). In particular, IL-17A and IL-17F can form homodimers (IL-17A/A or IL-17F/F) or heterodimers (IL-17A/F) (40–42).

Regarding their function, IL-17A and IL-17F are modest activators in terms of their pro-inflammatory potency when working alone (43), but they can dramatically amplify their signal by synergizing with other pro-inflammatory molecules, such as TNF-α, IL-1β, and IL-22 (31). The synergistic effect of IL-17A and IL-17F with TNFα has been shown in animal and human models in many cell types (31). Interestingly, the synergistic effects of IL-17A and IL-17F with TNFα or IL-1β are cell type-specific (31, 44). Although IL-17A has a more potent pro-inflammatory effect, IL-17F is found at higher levels (up to 30-fold) in lesional skin and serum of patients with PsO (45). These higher serum levels of IL-17F have also been observed in other IMIDs, such as PsA, radiographic axSpA or HS (46–49). Furthermore, lower mRNA levels of IL-17A have been found in PsA synovial tissue than in paired PsO skin samples (50). Indeed, IL-17A mRNA levels were 2.7-fold lower than those of IL-17F in the skin and 17.3-fold higher in synovial tissue, but IL-17A protein levels were 37.4-fold higher than those of IL-17F in synovial fluid (50). In rheumatoid arthritis, screening analysis of synovial fluid by multiplex ELISA showed higher protein levels of interleukin IL-23 and IL-17F in ectopic lymphoid neogenesis (ELN), an aggregation of T and B lymphocytes in nonlymphoid tissues, compared to ELN-negative samples (51). Moreover, downstream of IL-23, expression of CD21L (a marker selectively expressed in germinal center-containing synovial tissues) was significantly associated with IL-17F, IL-21, and IL-22, but not IL-17A in two independent sample sets of synovial tissue, supporting differential expression, and maybe function, of IL-17F and IL-17A in synovial tissue with different T/B organization (51).

**Figure 1:**
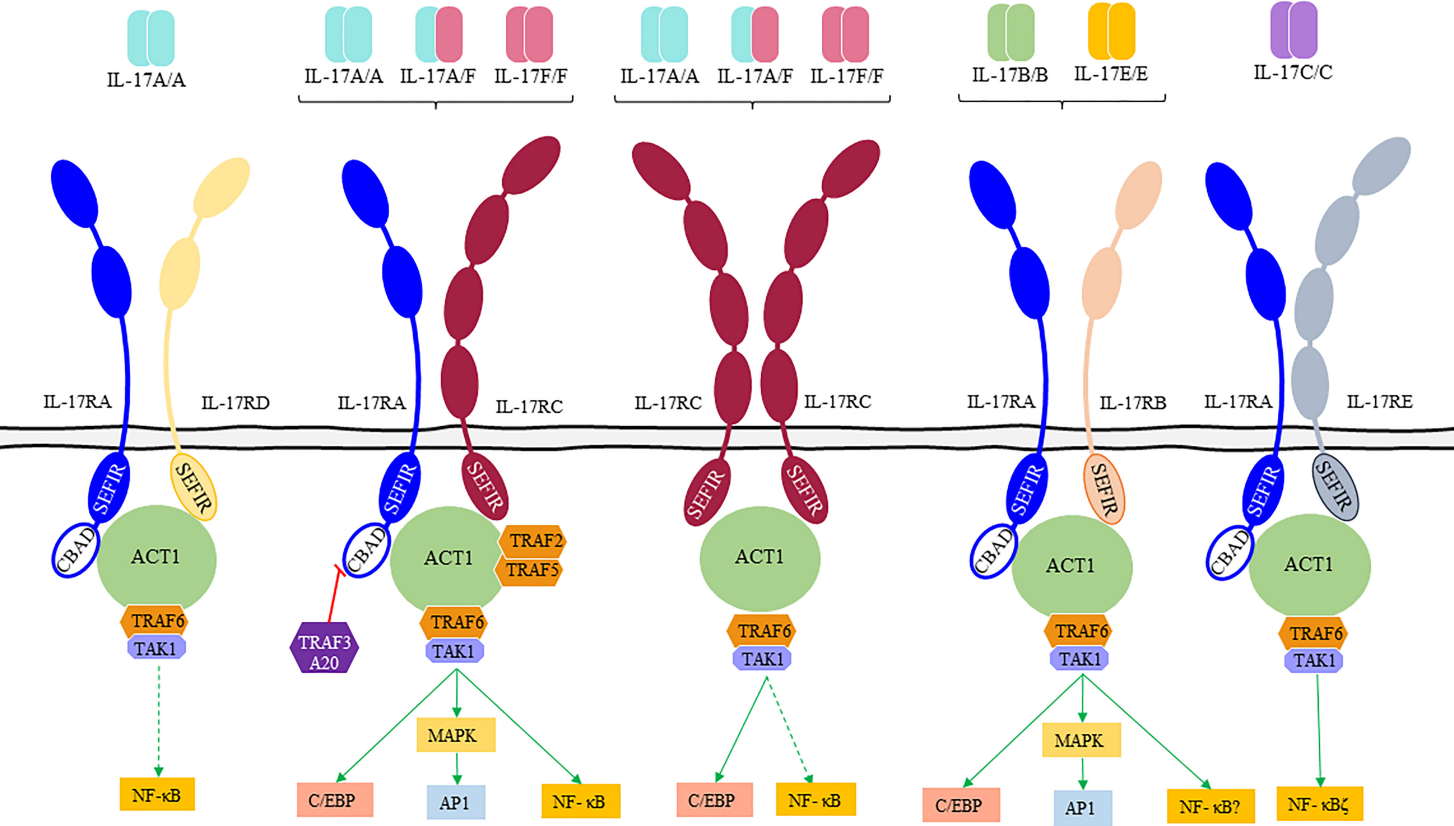
IL-17 receptor family (IL-17Rs), their ligands and downstream signaling pathways. IL-17Rs are classified in “short” and “tall” according to their extracellular domains (ECD). “Short” receptors (IL-17RA, IL-17RB, and IL-17RD) present a two-domains ECD and a disordered linker near the cytoplasmic membrane with a proline-rich motif, and “tall” receptors (IL-17RC and IL-17RE) have a larger ECD with two additional domains (52). IL-17A and IL-17F homodimers and IL-17A/IL-17F heterodimers bind to IL-17RA/C receptors leading to the recruitment of the nuclear factor-kappa B (NF-κB) activator 1 (Act1) by homotypic interactions between both SEFIR (similar expression to fibroblast growth factor genes) domains. Once the complex is formed other signaling molecules (TNF receptor-associated factor [TRAF]6, TRAF2, and TRAF5) are recruited to fully activate downstream signaling pathways. Bold arrows represent a preferential binding.

Signaling of IL-17s happens through a unique family of cell membrane receptors composed of five members (IL-17RA to IL-17RE, **Supplementary Table 2**, **Figure 1**). IL-17Rs can function either as homodimeric or heterodimeric proteins and are characterized by the presence of a conserved SEFIR (similar expression to fibroblast growth factor genes) domain in their intracellular region (53). Dimeric IL-17R complex induces binding of nuclear factor-kappa B (NF-κB) activator 1 (Act1) that functions as a linker with TRAF (TNF receptor-associated factor) family proteins. Recruitment of TRAF6 and transforming growth factor-β (TGF-β)-activated kinase 1 (TAK1) by Act1 leads to the activation of the classical NF-κB pathway (34, 52). In addition, IL-17s activate the CCAAT/enhancer-binding protein (C/EBP) family of transcription factors C/EBPβ and C/EBPδ regulating the expression of inflammation-related genes (IL-6, chemokines or IL-23R encoding genes) (54). Thus, the binding of Act1 to the IL-17R along with the binding of TRAF6 mediates the canonical pathway where C/EBP, AP-1, and NF-kB activation results in the transcription of inflammatory genes (1, 2, 55, 56). Also, Act1 interacts with the TRAF2/TRAF5 complex regulating the noncanonical pathway controlled by several RNA-binding proteins (such as HuR and Arid5a). TRAF3 can bind to the IL-17R preventing the formation of the IL-17R-Act1-TRAF6 complex. Moreover, TRAF4 can compete with TRAF6 for the binding site on Act1 blocking its signaling (56).

IL-17 receptors significantly differ in the composition of their extracellular domains, as revealed in the latest structural studies. IL-17RA is the only IL-17 chain known to contain a cytoplasmic domain named CBAD (C/EBP-β activation domain). This CBAD domain is part of a regulatory mechanism capable of coordinating the inhibition of IL-17s signaling through binding of TRAF3 to the ubiquitin-editing enzyme A20 (6). In addition to IL-17RA, both IL-17 A and F can signal as well through an IL-17RC:IL17RC homodimeric complex, debunking the long-standing concept that IL-17RA is a mandatory shared receptor (52). Mouse models have tested the functionality of the IL-17F/IL-17RC axis, showing that signaling through this axis can lead to a dysregulated inflammatory response (54, 57). This is mainly due to the inability of IL-17RC to bind to inhibitory regulatory partners such as A20 since the receptor lacks a CBAD domain (58, 59).

The functional patterns of IL-17RA and IL-17RC are not entirely overlapping (60), which might help to explain the tissue-specific roles observed of IL-17A and IL-17F. Blockade of IL-17RA could be potentiating an escape route *via* IL-17F/IL-17RC axis signaling (22, 57). Moreover, IL-17RA blockade leads to increase levels of circulating IL-17A in PsO that once the drug is withdrawn can readily signal through the IL-17RA/IL-17RC heterodimer inducing excessive IL-17A signaling (1).

**Figure 2:**
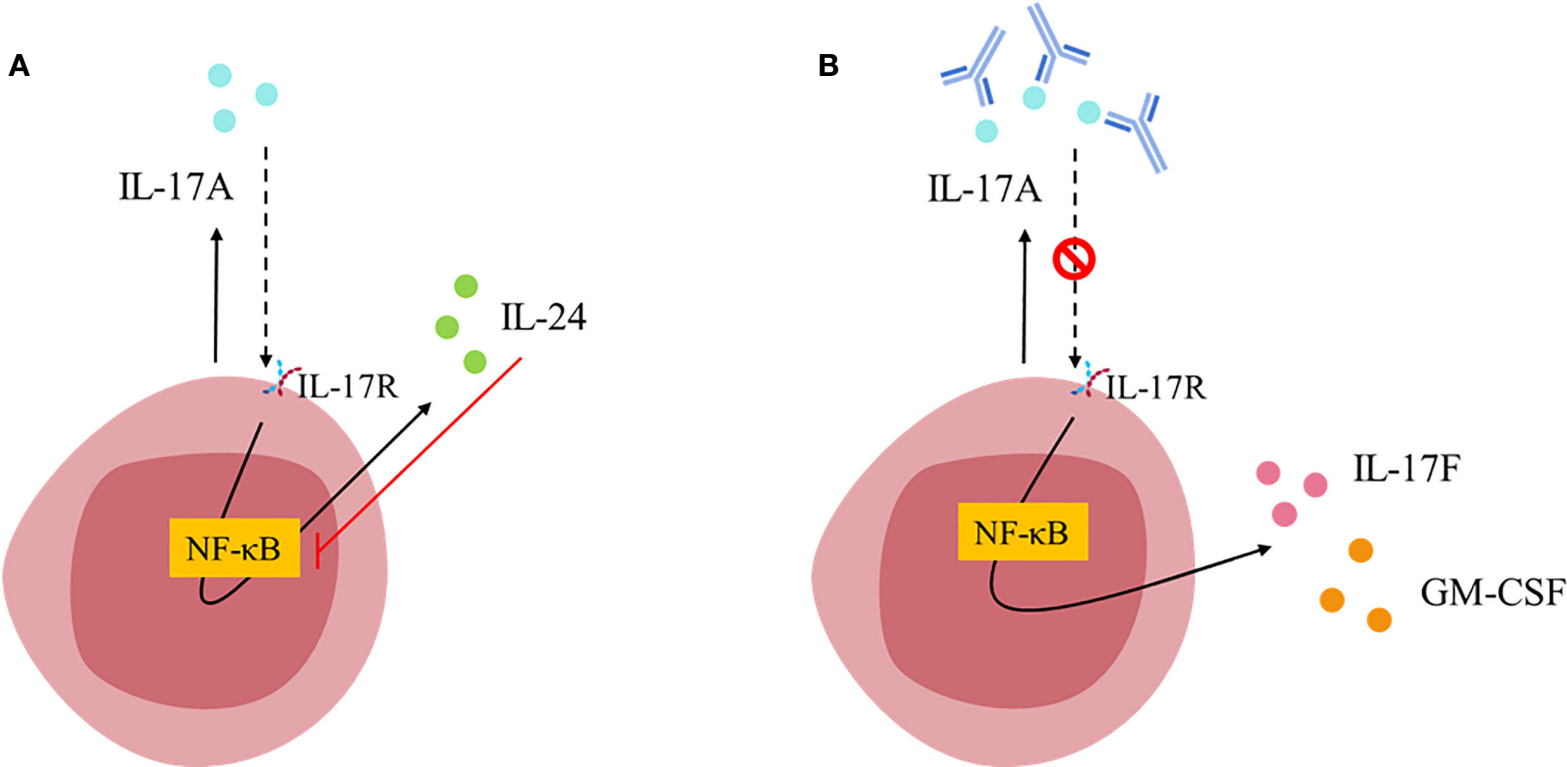
T_H_17 cell-intrinsic autocrine loop triggered by IL-17A [*Figure adapted from Chong et al., 2020* (61)]. **(A)** IL-17A binds to IL-17RA/IL-17RC receptor and activates NF-κB inducing the expression of IL-24, which in turn inhibits NF-κB leading to the repression of other T_H_17 signature cytokines (such as IL-17F and GM-CSF). **(B)** Blockade of IL-17A breaks the autocrine loop allowing NF-κB signaling and therefore, favoring IL-17F and GM-CSF expression.

Mechanistic studies have shown that immune cells, such as Th17 cells, are endowed with an autocrine regulatory feedback loop that tunes their pathogenicity (**Figure 2**) (61). IL-17A can bind in an autocrine manner to the IL-17RA : IL-17RC heterodimeric receptor, activates NF-κB and induces the secretion of IL-24. At the same time, IL-24 can also function in an autocrine manner and repress the expression of other Th17 signature cytokines including GM-CSF and IL-17F (61). Thus, blockade of IL-17A alone disrupts these autocrine pathways and unlocks the repressive role of IL-24, allowing for the release of GM-CSF and IL-17F and unlocking an unexpected pro-inflammatory escape route (**Figure 2**). This might explain the limitations of targeting IL-17A alone in some inflammatory conditions (62).

## 3 Cellular sources of IL-17A and IL-17F

**Figure 3:**
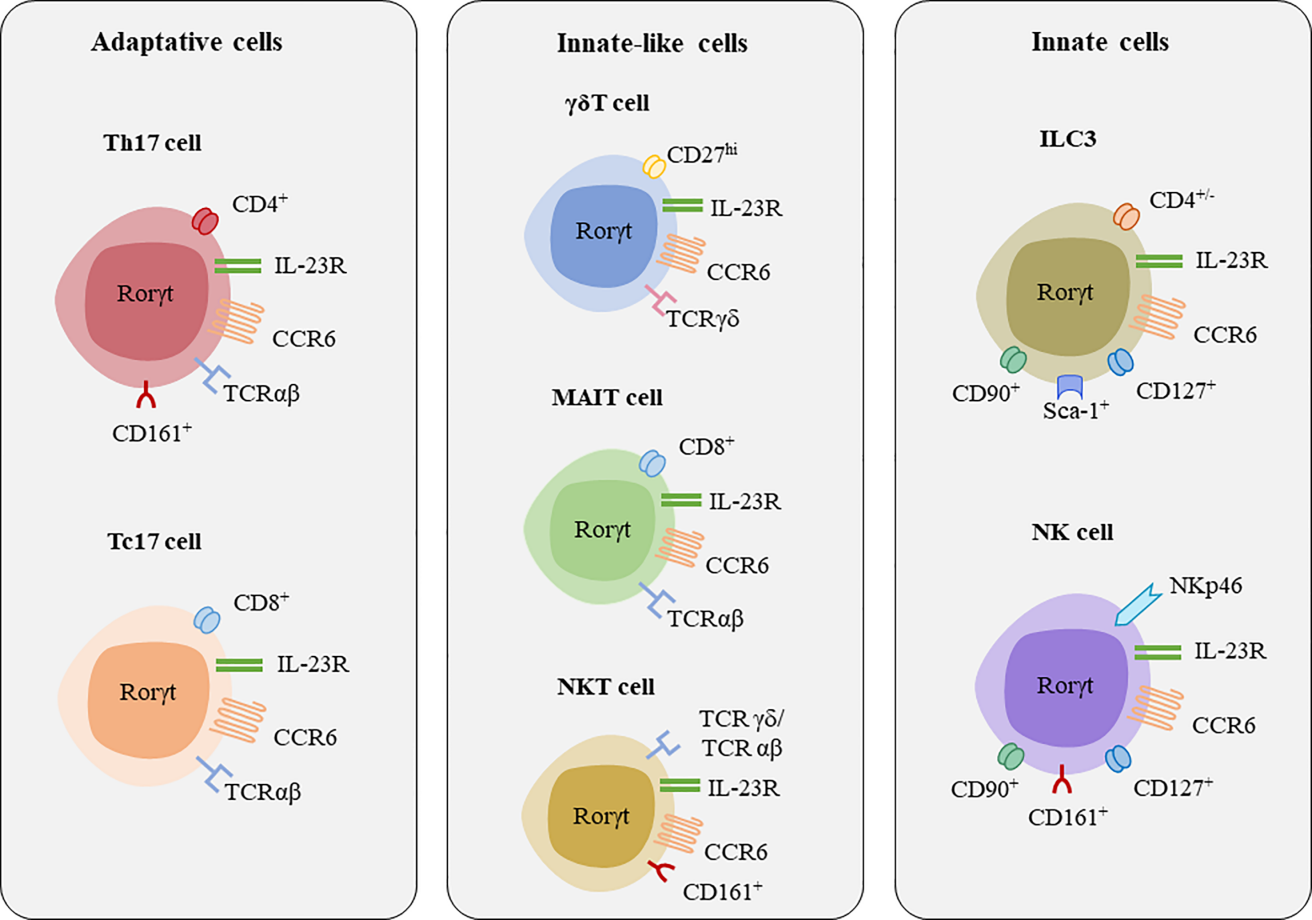
Major lymphocytes populations secreting IL-17A and IL-17F. [*Figure adapted from Veldhoen, 2017 *(60)]. Major transcription factor associated with these cells are RORγt, and some shared surface receptors include CCR6 and IL-23R.

As mentioned above, IL-17A and IL-17F are secreted by cells of the adaptative immune system such as Th17 cells and CD8^+^ cytotoxic T17 (Tc17) cells (63, 64), but also by innate immune cells such as group 3 innate lymphoid cells (ILC3s), and innate-like lymphocytes (ILLs) such as gamma delta (γδ) T, mucosal-associated invariant T (MAIT) cells, and natural killer T (NKT) cells (**Figure 3**, **Table 2**) (26, 64).

**Table 2:** Main characteristics of the immune cell populations secreting IL-17A and IL-17F and their role in autoimmune diseases.

	Adaptative cells		Innate-like cells			Innate cells	
	Th17	T_C_17	γδ T (γδ17)	MAIT	NKT	ILC3	NK
Role in PsO	Secretion of IL-17s that contribute to keratinocytes proliferation	Responsible of increased production of IL-17s in lesional skin	Contribute to IL-17A production In murine models are the primary source of IL-17s	Identified in skin and blood Proportion of IL-17A^+^ CD8^+^T cells in psoriatic plaques	NKT17 cells increased in PsO patients Decreased NKT2 vs. NKT17 ratio	Increased levels in blood and lesional and non-lesional skin	Present in inflammatory skin lesions
Role in PsA	Increased in blood and synovial fluid Possible contribution to bone erosion Increased number of Th17 cells compared to healthy controls	Increased frequency of CD8+ T cells Levels in peripheral blood correlates with the disease activity Enriched in synovial fluid of joints	Elevated levels found in synovial fluid of patients with active disease	Enriched in synovial fluid compared to peripheral blood	IL-17+ NKT cells in synovial fluid samples	Increased levels in blood and synovial fluid ILC2/ILC3 ratio correlated with disease activity scores, inflammation, and structural damage	Lower expression of NK cells in PsA patients than in rheumatoid arthritis patients
Role in SpA	Th17 cells dysregulation critical in the development of SpA Increased number of Th17 cells compared to healthy controls	Present hallmarks of tissue-resident memory cells in synovial fluid Present in gut, peripheral blood, and synovial fluid of patients (gut-joint migration axis)	In peripheral blood, a 3-fold higher frequency of circulating γδ T cells and 5-fold higher frequency of IL-23R-expressing γδ T cells *Ex vivo* experiments demonstrated are major sources of IL-17s in joints although they are not the most abundant cells	Higher proportion of IL-17+ MAIT cells in blood compared to healthy controls Increased MAIT cells in the synovial fluid of patients with AS	Increased in peripheral blood (respond to IL-23 stimulation) Possible protective role reducing joint inflammation Reduced in blood but increased in synovial fluid *Ex vivo* experiments demonstrated are major sources of IL-17s in joints although they are not the most abundant cells	Increased in the gut, peripheral blood, synovial fluid, and bone marrow of AS (migratory properties)	Increased IL-17s producing cells in peripheral blood (compared to healthy controls) IL-17s producing cells higher in serum fluid compared to peripheral blood
Role in enthesitis	Produce IL-17A upon stimulation	Produce IL-17A upon stimulation	Abundant in normal conditions in entheses Contribute to local IL-17A production independently of IL-23	Present in blood and in normal, unaffected entheses that contribute to IL-17A production		Normally expressed in soft tissue and bone adjacent to entheses. Local production of IL-17A	
Role in uveitis	Elevated levels in human peripheral blood and aqueous humor	Elevated CD8^+^CD146^+^ T_C_17 cells in Behçet’s disease and birdshot chorioretinopathy Dysregulated cytokine expression in acute anterior uveitis (increased GM-CSF, IL-17A and IL-17F secretion compared to healthy controls or patients with SpA)	Responsible of the IL-17 increase in peripheral blood and in the aqueous humour	Increased IL-17A and IL-17F production in acute anterior uveitis	Elevated levels in peripheral blood and aqueous humour in Behçet’s uveitis In mice model of autoimmune uveitis, NKT function seemed to correlate with susceptibility		Increased levels in eyes and spleen of experimental autoimmune uveitis model Behçet’s disease patients have an increased number of cytokine secreting NK cells
Role in IBD	Increased IL-17A expression	Enrichment of T_c_17 cells with proinflammatory features (CD6 expression) in active Crohn’s disease	Increased IL-17A expression Primary source of IL-17A	*In vitro* IBD models, MAIT cells produced more IL-17 Reduced circulating MAIT cell frequencies and increased IL-17 production in ulcerative colitis patients	Reduction in IBD patients Protective role (reducing gut inflammation) in IBD animal models	Increased IL-17A expression	Express IL-17F under physiological conditions
References	(24, 65–71)	(68–82)	(29, 63, 68, 71, 79, 83–89)	(64, 80, 90–97)	(64, 68, 86, 88, 98–103)	(24, 68, 71, 86, 104–109)	(110–113)

AS, ankylosing spondylitis; GM-CSF, Granulocyte Macrophage Colony-Stimulating Factor; IBD, inflammatory bowel disease; IL-17, interleukin 17; ILC3, group 3 innate lymphoid cells; MAIT, mucosal associated invariant T cells; NK, natural killer cells; NKT, natural killer T cells; PsA, psoriasis arthritis; PsO, psoriasis; SpA, spondyloarthritis; Th17, CD4^+^ helper T cells; Tc17, CD8^+^ cytotoxic T17.

### 3.1 Th17 and Tc17 cells, the conventional IL-23-dependent producers of IL-17A and IL-17F

**Figure 4:**
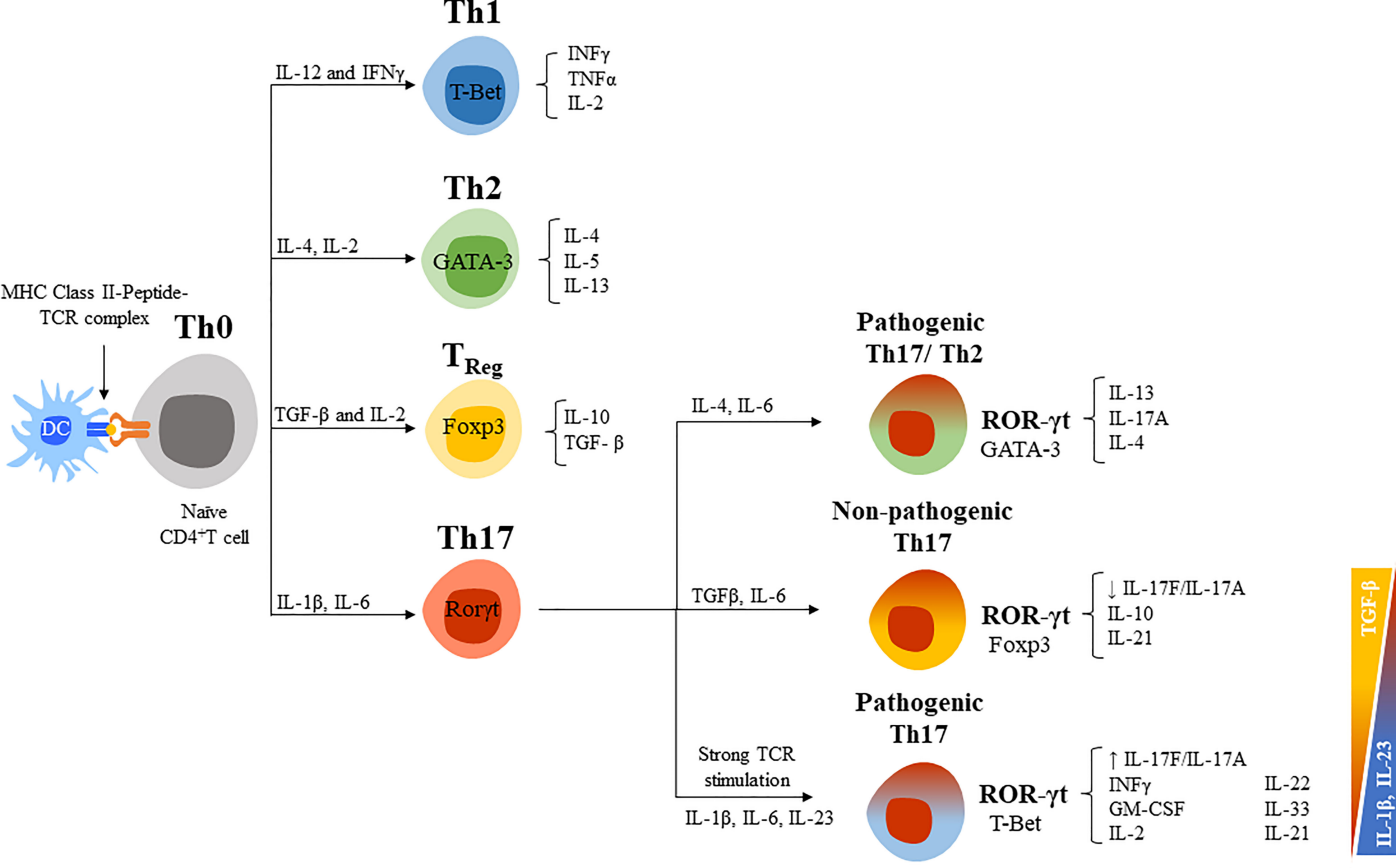
CD4+ helper T cells differentiation routes. [*Figure adapted from Ruiz de Morales et al., 2020* (5)]. CD4^+^ helper T cells (Th0) differentiate into effector T cell subsets (Th1, Th2, or Th17) and regulatory T cells (T_Reg_) with specific signal transduction mechanisms, transcription factors, and cytokine profiles for each cell lineage. IL-12 and IFNγ are critical cytokines initiating the downstream signaling cascade to develop Th1 cells, the T-box transcription factor (T-bet) is the master regulator for its differentiation, and mainly secrete IFNγ and IL-2 (116). For Th2 lineage development, IL-4 and IL-2 are crucial cytokines and GATA3 is the master regulator transcription factor. Among their key effector cytokines are IL-4, IL-5, and IL-13. T_Reg_ cells are generated after antigen stimulation under TGF-β and IL-2 presence, express the Foxp3 transcription factor and have a role in maintaining immune homeostasis through their main effector cytokines TGF-β and IL-10. IL-1β, IL-6, or IL-21 are required for the differentiation of Th17 cells, which express RORgt and secrete IL-17s (115, 117). TGF-β and IL-6 exposure sustained the differentiation of non-pathogenic Th17 cells (co-expression of T-Bet), whereas IL-23 induce a pathogenic profile (co-expression of Foxp3). Newly described Th17/Th2 cells express markers of both CD4^+^ T cells (CD161 and RORγt, and GATA3) and produce IL-4 and IL-17. This phenotype is also considered pathogenic (118).

Th17 cells are the main responders against extracellular pathogens such as fungi and bacteria. They are a well-studied source of IL-17A and IL-17F, but they can also secrete other cytokines such as IL-21, IL-22, IL-24, and GM-CSF (114, 115). The differentiation of naïve CD4^+^ helper T cells (Th0) into effector subsets (Th1, Th2, or Th17) or regulatory T cells (T_reg_) depends on the local cytokine milieu (**Figure 4**) (117). T-bet (T-box expressed in T cells), GATA3 (GATA binding protein 3) and Foxp3 (forkhead box P3) are the main transcription factors expressed by Th1, Th2, and T_reg_ cells, respectively, and are responsible for the lineage-specific cytokine profiles of each subset. Th1 are defined by their preferential production of IL-2 and IFNγ and respond mostly against intracellular pathogens (bacteria, virus, etc.); Th2 for IL-4, IL-5, and IL-13 and respond against parasites and allergens; and T_reg_ for TGF-β and IL-10 and are involved in immune tolerance and lymphocyte homeostasis. Conversely, retinoid-related orphan receptor gamma t (RORγt) is the hallmark transcription factor that determines the differentiation of Th17 cells, inducing the production of IL-17A and IL-17F, and surface molecules such as the C-C chemokine receptor type 6 (CCR6) and IL-23R (119, 120). In general, it has been established that IL-1β and IL-6 are required for the differentiation of Th17 cells, whereas TGF-β and IL-23 favor lineage expansion, maintenance, and survival (121, 122). However, the balance between IL-23 and TGF-β prompts a different cytokine profile of Th17 cells and their pathogenicity (**Figure 4**) (123, 124). An increase in the TGF-β/IL-23 ratio can result in the generation of non-pathogenic Th17 cells, whereas a reduction in this ratio converts them to pathogenic Th17 cells (123, 125, 126). The lack of IL-10 expression and enhanced GM-CSF in Th17 cells has been also associated with a pathogenic phenotype (125). The pro-inflammatory mediator prostaglandin E2 (PEG2) is another potent activator of Th17 cells that induces a pathogenic phenotype (increasing RORγt and IL-17A and reducing IL-10) (127). Indeed, PEG2 and cyclooxygenase 2 (COX2) are increased in RA synovial tissue and, in mice models, PGE2 modulates the severity of the disease.

Activated Th17 cells also differ in terms of the subtype of IL-17s that they preferentially express, differentiating between subpopulations that express IL-17A, IL-17A/F, or IL-17F. Three factors have been observed to control this differentiation, the cytokine environment, the strength or concentration of antigenic signaling through the T-cell receptor (TCR), and the duration of the stimulus (47, 128). Low-strength T cell activation preferentially promotes the induction of the IL-17A^+^ subpopulation of Th17 cells whereas high-strength stimulation favors the IL-17F^+^ subpopulation. Furthermore, IL-17A^+^ and IL-17F^+^ Th17 cells also differ in terms of the cytokine profiles that they produce. IL-17F^+^ cells have been associated with a more pathogenic phenotype in inflammatory diseases since they express reduced levels of IL-10 and GM-CSF and a higher level of IFN-γ compared to IL-17A^+^ cells (47). Moreover, the expression of IL-17A and IL-17F is differentially regulated over time. IL-17A is rapidly produced upon T-cells stimulation, whereas IL-17F expression shows a gradual increase with higher levels at later stages of activation. Conversely, unlike IL-17F, IL-17A expression is not sustained by continuous activation of T-cells (129). This might suggest that, whereas IL-17 A has an important role upon inflammation onset, IL-17F would acquire more relevance in its chronification. This would explain why in certain cases targeting IL-17A alone might not be enough for a long-term disease control (13–16, 61), but dual inhibition of IL-17 A and F might be of choice.

CD8^+^ cytotoxic T cells (Tc17) are another subset of cells from the adaptative immune system that produce IL-17A, IL-17F, IL-21, IL-22 and express RORγt (130). Like Th17 cells, cytokines IL-6 or IL-21 along with TGF-β determine the differentiation of Tc17 cells. In addition, IL-23 stabilizes their phenotype. Tc17 cells produce small amounts of IFNγ, granzyme and perforin, exerting a low cytotoxicity (131–133).

### 3.2 Innate lymphoid cells and innate-like lymphocytes: additional sources of IL-17A and IL-17F

Innate lymphoid cells (ILCs) and innate-like lymphocytes (ILLs such as ILC3, γδ T cells, MAIT cells, and NKT cells) are additional sources of IL-17A and IL-17F that have important roles in controlling homeostasis and protecting against infections (134–138). However, the dysregulation of these cells promotes inflammatory responses contributing to the pathogenesis of inflammatory diseases such as PsO, PsA, axSpA, and IBD (**Table 1**) (90, 116, 139–141). The innate nature of these cells allows them to be rapidly activated during early phases of inflammatory responses and become major sources of IL-17s through a restricted TCR engagement or in response to certain cytokine environments (91, 135). Several studies have highlighted the complexity and number of cytokines that can induce the production of IL-17A and IL-17F by innate and innate-like lymphocytes in response to other cytokines, different from the canonical IL-23, such as IL-7 (45, 141, 142), IL-9 (143), IL-12, IL-1β and IL-18 (28). This is consistent with data obtained in inflammatory diseases (PsO, PsA, axSpA, and HS) where IL-17F levels are higher than those of IL-17A (104, 144–146). Recent evidence has shown a trend in ILC3s, γδ T, and MAIT cells to produce predominantly IL-17F in a mode independent of IL-23 (28, 116). Production of IL-17s in ILCs and ILLs is also dependent on RORγt expression, and although these cells can present the cell surface marker IL-23R, they can also follow an IL-23-independent pathway (91, 142). One plausible explanation could be a molecular disconnection: IL-23 binds to the receptor but cannot activate IL-17s production due to a lower expression of tyrosine kinase 2 (TYK2) and signal transducer and activator of transcription 3 (STAT3), as some transcriptional studies seem to point out (91).

ILC3s, predominantly found in mucosal tissues and skin, are characterized for presenting an invariant TCR, expressing RORγt and therefore producing IL-17A, IL-17F and/or IL-22. Interestingly, *in vitro* studies performed with human samples have shown that ILC3s cells can be induced to produce IL-17A and IL-17F, in an IL-23 independent manner, upon exposure to a combination of IL-1β and IL-2 (28).

γδ T cells comprise 50% of the intraepithelial lymphocyte cells in mucosal and epithelial tissues and 3-5% of all blood lymphoid cells (135, 136). They are atypical T cells characterized by the expression of a semi-invariant γδ T cell receptor (TCR) that can recognize a broad range of microbial antigens. Most of the antigens and the recognition mechanism of γδ T cells are still unknown, binding to phosphorylated metabolites such as microbial (E)-4-Hydroxy-3-methyl-but-2-enyl pyrophosphate (HMB-PP) or eukaryotic isoprenoid precursor (IPP), or even to lipid antigens presented by cluster of differentiation 1 (CD1) molecules has been reported (135, 136). The γδ17 subset expresses RORγt and share many common features with Th17 cells (cell surface expression of IL-23R, CCR6, CCR2 and CXCR6) (147, 148). Secretion of IL-17A and IL-17F by γδ17 cells can take place upon IL-23 stimulation (138, 149), or in an IL-23-independent manner in the presence of other cytokines such as IL-7, or combined IL-12 and IL-18 stimulation (28, 83, 84, 135).

MAIT cells are predominantly CD8^+^ T cells and display rapid innate-like effector functions upon activation. These cells express an invariant TCR that recognizes small metabolites derived from the microbial vitamin B2 (riboflavin) biosynthesis and are restricted by MHC-related molecule-1 (MR1) (85). Overall, MAIT cells are abundant in humans and can be found in many tissues with varying frequency such as blood (up to 10% of T cells), liver (up to 20 to 50% of T cells), synovial tissue (~5%), intestine (1.5-4% CD3^+^ T cells), lungs (3%) and skin (0.5-2%) (72, 90, 92, 93, 150–152). Expression of RORγt polarises MAIT cells towards a Th17-like phenotype including the production of IL-17A and IL-17F cytokines, and receptors such as IL-1R, IL-7R, IL-12R, IL-18R and IL-23R (90, 94). *In vitro* studies of MAIT cells isolated from human blood samples have shown that exposure to a combination of IL-12 and IL-18 can induce the production of IL-17A and IL-17F independently of IL-23. Interestingly, strong TCR stimulation in the presence of IL-12 and IL-18 can influence the cytokine profile of MAIT cells with a bias towards IL-17F (28).

NKT cells have features of both NK cells and T cells (153). Three subpopulations can be distinguished based on the transcription factors and cytokine profiles that they express, namely analogous to Th1 (NKT1), Th2 (NKT2) and Th17 cells (NKT17) (152). NKT17 cells are mainly present in lymph nodes, skin, and lungs, and their survival and expression depend on IL-7 (98, 142, 154). NKT17 cells secrete Th17-related cytokines, such as IL-17A, IL-17F, IL-21 and IL-22 (155, 156) and express distinctive markers of Th17 cells such as IL-1R, IL-23R, CCR6, CD103, and CD138 (157–159). Apart from secreting IL-17A and IL-17F in an IL-23-dependent manner (160, 161), NKT can also produce IL-17s after stimulation with TGF-β and IL-1β (162). CD161 (or NK1.1 in murine models) is usually expressed on NK cells and associated with the inhibition of their function (163). The expression of CD161 is regulated by RORγt; it is a marker of IL-17 producing T cell subsets, including CD4^+^ and CD8^+^ T cells, and some populations of T_reg_ cells besides NKT cells.

## 4 Conclusion

IL-17A and IL-17F have relevant physiological roles and their dysregulation can result in pathological conditions. Abnormal levels of these cytokines have been found in IMIDs, making them potential therapeutic targets. Adaptive Th17 cells are generally considered the main producers of IL-17s, and IL-23 was assumed to be indispensable to regulate their secretion. However, current evidence proves that other innate and innate-like cells can secrete IL-17A and IL-17F triggering different signaling pathways, which can be IL-23-independent. The crosstalk between IL-17s and IL-23 in autoimmune and inflammatory diseases is widely recognized, and the IL-23/IL-17 axis has been targeted in the development of therapeutic agents. Inhibitors of IL-17A, IL-17F, or IL-23 have promising results, although they do not yield the same clinical effect in all IMIDs. The evidence of a non-linear relationship between IL-23 and IL-17s can underly these tissue-specific functions of IL-17A and IL-17F.

Therefore, IL-23-independent signaling pathways and additional sources of IL-17A and IL-17F, apart from the adaptative immune cells, constitute alternative processes underpinning pathological conditions. The present review explores the recent literature regarding IL-17s’ alternative sources and signaling pathways independent of IL-23. The types of IL-17R are also important to modulate the response in different tissues. We hope this review may contribute to highlight the importance of considering all IL-17A and IL-17F cellular sources and alternative signaling pathways in designing new therapies and improving treatment selection for IMIDs.

## Author contributions

All authors equally contributed to the literature review, drafting and critical revision of the manuscript. All authors contributed to the article and approved the submitted version.
