# Corrigendum: The paradigm of IL-23-independent production of IL-17F and IL-17A and their role in chronic inflammatory diseases

**DOI:** 10.3389/fimmu.2023.1332177

**Published:** 2023-11-22

**Authors:** Victoria Navarro-Compán, Luis Puig, Silvia Vidal, Julio Ramírez, Mar Llamas-Velasco, Cristina Fernández-Carballido, Raquel Almodóvar, José Antonio Pinto, Eva Galíndez-Aguirregoikoa, Pedro Zarco, Beatriz Joven, Jordi Gratacós, Xavier Juanola, Ricardo Blanco, Salvador Arias-Santiago, Jesús Sanz Sanz, Rubén Queiro, Juan D. Cañete

**Affiliations:** ^1^ Department of Rheumatology, Hospital Universitario La Paz, IdiPaz, Madrid, Spain; ^2^ Department of Dermatology, Hospital de la Santa Creu i Sant Pau, Barcelona, Spain; ^3^ Immunology-Inflammatory Diseases, Institut de Recerca de l’Hospital de la Santa Creu i Sant Pau, Biomedical Research Institute Sant Pau (IIB Sant Pau), Barcelona, Spain; ^4^ Arthritis Unit, Department of Rheumatology, Hospital Clínic and Instituto de Investigaciones Biomédicas August Pi i Sunyer (IDIBAPS), Barcelona, Spain; ^5^ Department of Dermatology, Hospital Universitario La Princesa, Madrid, Spain; ^6^ Department of Rheumatology, Hospital Universitario San Juan de Alicante, Alicante, Spain; ^7^ Department of Rheumatology, Hospital Universitario Fundación Alcorcón, Alcorcón, Madrid, Spain; ^8^ Department of Rheumatology, Complejo Hospitalario Universitario de A Coruña, Instituto de Investigación Biomédica de A Coruña (INIBIC), A Coruña, Spain; ^9^ Department of Rheumatology, Hospital Universitario de Basurto, Bilbao, Spain; ^10^ Department of Rheumatology, Hospital Universitario 12 de Octubre, Madrid, Spain; ^11^ Department of Rheumatology, Medicine Department Autonomus University of Barcelona (UAB), I3PT, University Hospital Parc Taulí Sabadell, Barcelona, Spain; ^12^ Department of Rheumatology, University Hospital Bellvitge, Instituto de Investigación Biomédica de Bellvitge (IDIBELL), Barcelona, Spain; ^13^ Department of Rheumatology, Hospital Universitario Marqués de Valdecilla, Instituto de Investigación Marqués de Valdecilla (IDIVAL), Santander, Spain; ^14^ Department of Dermatology, Hospital Universitario Virgen de las Nieves, Granada, Spain; ^15^ Instituto de Investigación Biosanitaria ibs.GRANADA, Granada, Spain; ^16^ Department of Dermatology, Facultad de Medicina, Universidad de Granada, Granada, Spain; ^17^ Department of Rheumatology, Hospital Universitario Puerta del Hierro Majadahonda, Madrid, Spain; ^18^ Department of Rheumatology, Hospital Universitario Central de Asturias, Oviedo, Asturias, Spain

**Keywords:** IL-17A, IL-17F, IL-23, spondyloarthritis, Th17 cells, MAIT cells, γδ T cells, psoriasis

In the published article, there was an error in **Table 1** as published. The cell corresponding to ixekizumab in HS must be yellow rather than green, since it is not approved for that indication, nor has phase 3 clinical development been published.

Also, there is no data regarding clinical studies using tildrakizumab for IBD, so the color has been changed to yellow.

The legend has been modified for clarification. The changes include the modification from “Green: Shown efficacy” to “Green: approved or shown efficacy in phase 3 clinical trials”.

The corrected **Table 1** and its caption “Available therapies targeting IL-17 and IL-23 in IMIDs” appear below.

**Table 1:** Available therapies targeting IL-17 and IL-23 in IMIDs.

Mechanism of action	Therapy	Approved indications*	PsO	PsA	axSpA	HS	IBD	Uveitis
Anti-IL-17A	Ixekizumab	Plaque psoriasis, psoriatic arthritis, axial spondylarthritis						
	Secukinumab	Plaque psoriasis, psoriatic arthritis, axial spondylarthritis, juvenile idiopathic arthritis						
Anti-IL-17RA	Brodalumab	Plaque psoriasis						
Anti-IL-17A and IL-17F	Bimekizumab	Plaque psoriasis, psoriatic arthritis, and axial spondylarthritis. Phase 3 clinical development completed for hidradenitis suppurativa, EMA approval pending						
Anti-IL12/23	Ustekinumab	Plaque psoriasis, psoriatic arthritis, Crohn’s disease, ulcerative colitis						
Anti-IL23p19	Guselkumab	Plaque psoriasis, psoriatic arthritis. In phase 3 clinical development for Crohn’s disease and ulcerative colitis						
	Risankizumab	Plaque psoriasis, psoriatic arthritis, Crohn’s disease. In phase 3 clinical development for ulcerative colitis						
	Tildrakizumab	Plaque psoriasis. In phase 3 clinical development for psoriatic arthritis						

axSpA, axial spondyloarthritis; HS, hidradenitis suppurativa; IBD, inflammatory bowel disease; IL, interleukin; PsA, psoriasis arthritis; PsO, psoriasis.

*The table only includes therapies that have been approved by the European Medicines Agency (EMA) or that have published phase 3 clinical development.

Green: approved or shown efficacy in phase 3 clinical trials. Red: Lack of efficacy. Yellow: Insufficient or unclear evidence.

The authors apologize for this error and state that this does not change the scientific conclusions of the article in any way. The original article has been updated.

