# Supplementary material for: The paradigm of IL-23-independent production of IL-17F and IL-17A and their role in chronic inflammatory diseases
Source: Front Immunol. 2023 Aug 4;14:1191782. doi: 10.3389/fimmu.2023.1191782 (PMC10437113; doi:10.3389/fimmu.2023.1191782)
Supplement: Supplementary file 1 [file Table_1.docx]

Supplementary Material

The paradigm of IL-23-independent production of IL-17F and IL-17A and their role in chronic inflammatory diseases

**Navarro-Compán V^1^*, Puig L^2^*, Vidal S^3^, Ramírez J^4^, Llamas-Velasco M^5^, Fernández-Carballido C^6^, Almodóvar R^7^, Pinto JA^8^, Galíndez-Agirregoikoa E^9^, Zarco P^7^, Joven B^10^, Gratacós J^11^, Juanola X^12^, Blanco R^13^, Arias S^14^, Sanz Sanz J^15^, Queiro R^16#^, Cañete JD^4#^.**

**^#^ Correspondence:** Juan D. Cañete, e-mail: [jcanete@clinic.cat](mailto:jcanete@clinic.cat); and Rubén Quiero, e-mail: [rubenque7@yahoo.es](mailto:rubenque7@yahoo.es)

**Supplementary Table 1.** Interleukin 17 (IL-17) family members, gene location and their receptors.

| **Cytokine** | **Chromosome** | **Receptor(s)^1^** | **PDB Accession Number^2^** | **Cellular sources^3,4^** | **Described functions^3,4^** |
| --- | --- | --- | --- | --- | --- |
| IL-17A | 6p12 | IL-17RA:IL-17RC  IL-17RC:IL-17RC  IL-17RA:IL-17RD | 2VXS  5HHX, 5HHV  5HI5, 5HI4, 5HI3  5VB9  4HR9, 4HSA | Th17, Tc17, γδ T cells, MAIT cells, NK cells, NKT cells, LTi, ILC3, neutrophils, epithelial cells, keratinocytes, fibroblasts | - Pro-inflammatory responses. - Protective role at barrier surfaces (skin and mucosal tissues) against extracellular bacterial and fungal infections. - Wound healing. - Pathogenic roles in immune-mediated inflammatory diseases and in some cancers. |
| IL-17F | 6p12 | IL-17RA:IL-17RC  IL-17RC:IL-17RC | 1JPY  6HGO, 6HG9, 6HG4,  6PPG |  |  |
| IL-17A/F | 6p12 | IL-17RA:IL-17RC  IL-17RC:IL-17RC | 5N92, 5NAN |  |  |
| IL-17B | 5q32-34 | IL-17RA:IL-17RB | N/A | Neutrophils, B cells, neurons, stromal cells, epithelial cells, chondrocytes | - Pro-inflammatory role (increase during intestinal inflammation, promotion of neutrophil migration). - Anti-inflammatory role (blockade of IL-25 signaling during mucosal inflammation. - Pathogenic role: involved in inflammatory arthritis, gastric and breast cancers, lung fibrosis, and protective role in colitis and asthma. |
| IL-17C | 16g24 | IL-17RA:IL-17RE | N/A | Keratinocytes, lung epithelial cells, colon epithelial cells, cutaneous neurons | - Anti-microbial protective response. - Barrier maintenance in skin and intestine (produced by epithelial cells). - Protection of peripheral sensory neurons promoting survival and growth to replace damaged nerves. |
| IL-17D | 13q12.11 | ND | N/A | Skeletal muscle, brain, adipose, heart and lung tissue | - Increase during viral infections and in tumours - Pro-inflammatory cytokine response through IL-6, IL-8 and GM-CSF. - Pathogenic role in sepsis and certain intracellular infections. |
| IL-17E (IL-25) | 14q11.2 | IL-17RA:IL-17RB | N/A | Th2 cells, Th9 cells, fibroblasts, endothelial cells, macrophages, iNKT, ILC2, intraepithelial lymphocytes, lung epithelial cells, cells of the gastrointestinal tract and uterus, eosinophils, basophils, mast cells, keratinocytes | - Associated with type 2 immunity (induces expression of IL-4, IL-5, IL-13, and TSLP) - Promotion of epithelial cell hyperplasia - Increase mucus secretion and airway hyperreactivity. - Inhibit Th17-mediated autoimmunity. - Pathogenic role in in allergy and psoriasis. |

γδ T cells: gamma delta T cells; ILC: innate-like lymphocytes; iNKT: invariant natural killer T cells; LTi: Lymphoid tissue inducer; MAIT: mucosal-associated invariant T cells; NK: natural killer cells; NKT: natural killer T cells; Tc17: CD8^+^ cytotoxic T17 cells; Th: T helper cells.

^1^Ligand receptor-paring has not been assessed for all members.

^2^Crystal structures of all members are not available.

^3^Information extracted from McGeachy et al. Immunity 2019; 50(4):892-906.

^4^Information extracted from Fletcher JM, et al. Clin Exp Immunol. 2020 May 7.

**Supplementary Table 2.** Interleukin 17 (IL-17) receptors, gene location and expression pattern.

| **Receptor** | **Genetic location** | **Exons** | **Receptor type** | **Expression pattern** | **PDB Accession Number** |
| --- | --- | --- | --- | --- | --- |
| IL-17RA | 22q11.1 | 13 | Short | Ubiquitous expression in tissues. Relatively higher expression in haematopoietic tissues | 4NUX  5NAN  4HSA  5HHX, 5HHV  3JVF |
| IL-17RB | 3p21.1 | 11 | Short | Various endocrine tissues, kidney, liver and T_H_2 cells | 3VBC |
| IL-17RC | 3p25.3 | 19 | Tall | Expressed at high levels in joints and glandular tissues, such as the adrenal gland, prostate, liver, and thyroid. Low levels of expression in hematopoietic tissues | N/D |
| IL-17RD | 3p21.2 | 13 | Short | Expressed in epithelial cells of breast, prostate, thyroid gland, ovarian surface and endothelial cells | N/D |
| IL-17RE | 3p25.3 | 16 | Tall | Not defined | 6HGA, 6HG9, 6HG4 |
